# Critical update, systematic review, and meta‐analysis of oral erythroplakia as an oral potentially malignant disorder

**DOI:** 10.1111/jop.13304

**Published:** 2022-05-12

**Authors:** Alejandro I. Lorenzo‐Pouso, Irene Lafuente‐Ibáñez de Mendoza, Mario Pérez‐Sayáns, Alba Pérez‐Jardón, Cintia M Chamorro‐Petronacci, Andrés Blanco‐Carrión, José Manuel Aguirre‐Urízar

**Affiliations:** ^1^ Oral Medicine, Oral Surgery and Implantology Unit (MedOralRes Group) Faculty of Medicine and Dentistry, University of Santiago de Compostela A Coruña Spain; ^2^ Oral Medicine and Oral Pathology, Department of Stomatology II University of the Basque Country/EHU Leioa Spain; ^3^ Health Research Institute of Santiago de Compostela (IDIS), ORALRES group Universidade de Santiago de Compostela Santiago de Compostela Spain

**Keywords:** malignant development, meta‐analysis, mouth neoplasm, oral erythroplakia, oral potentially malignant disorder

## Abstract

**Background:**

Oral erythroplakia has been classically considered as the potentially malignant disorder with the highest rate of malignant development into squamous cell carcinoma. This critical systematic review and meta‐analysis aim to estimate the malignant development rate of oral erythroplakia and identify the associated risk factors.

**Methods:**

We performed a bibliographic search in PubMed, Scopus, Web of Science, Embase, and LILACS, with keywords “erythroplakia,” “erythroplasia,” “malignant transformation,” “malignant development,” “malignization,” “carcinogenesis,” “oral cancer,” “oral squamous cell carcinoma,” “mouth neoplasm,” and “prognosis.” Meta‐analysis was conducted using a random‐effects model.

**Results:**

Ten observational studies with 441 patients met the inclusion criteria, whose mean malignant development rate was 12.7% and with a mean follow‐up period of patients of 6.66 years. In the initial biopsy, 42.8% of oral erythroplakia were already squamous cell carcinoma. The buccal mucosa was the most frequent location of oral erythroplakia, but the floor of the mouth was the most common site of malignant development. All patients who underwent malignant development showed epithelial dysplasia on the initial diagnostic biopsy.

**Conclusion:**

Overall malignant development rate of OE in the meta‐analysis was 19.9%. We could not associate any specific clinicopathological feature with the malignant development. The presence of epithelial dysplasia in the initial biopsy remains the worst prognostic factor. Further observational studies on OE are needed, with well‐established diagnostic criteria and good clinical follow‐up, in order to identify the true risk of malignant development of oral erythroplakia and the related risk factors.

## 1 INTRODUCTION

Oral erythroplakia (OE) is a rare but highly risky oral potentially malignant disorder (OPMD) that has been the subject of multiple controversies since its initial reports.^1^, ^2^, ^3^, ^4^, ^5^ OE is classically considered as the OPMD with the highest rate of malignant development (MD).^6^, ^7^, ^8^, ^9^ According to the last consensus convened by the WHO Collaborating Centre for Oral Cancer,^9^ OE represents the red counterpart of oral leukoplakia (OL), and it is defined as “a predominantly fiery red patch that cannot be characterized clinically or pathologically as any other definable disease.”

Due to its clinical appearance, it is very important to perform a good differential diagnosis of this pathology with other OPMD like erythroleukoplakia and oral lichen planus, and other “red disorders” of the oral mucosa, such as candidiasis, lupus, and fixed drug eruptions.^6^, ^9^, ^10^ Unfortunately, there are not enough data on the different clinicopathological factors that might influence the MD of OE, including age and gender of patients, tobacco, alcohol, or betel consumption, location of lesions, and presence of epithelial dysplasia (ED).

With this background, we designed this critical systematic review and meta‐analysis with all the available scientific evidence related to the MD of OE in order to estimate its MD rate and discuss the associated risk factors.

## 2 MATERIALS AND METHODS

### 2.1 Protocol and registration

This systematic review and meta‐analysis comply with PRISMA^11^ and MOOSE guidelines.^12^ The review protocol was submitted to the PROSPERO register (CRD42022299026) to minimize risk of bias and improve the transparency, precision, and integrity. An ad hoc review group was created with oral medicine specialists of the Oral Medicine Unit from the University of Santiago de Compostela (MPS, AILP, and APJ) and the Oral Medicine and Pathology Unit of the University of the Basque Country (JMAU and ILIM).

### 2.2 Focused questions

This review was designed to answer the following questions: (1) what is the overall MD of OE? and (2) what are the risk factors contributing to the MD of OE? We used the PECOS acronym as follows: (1) patients with OE (population), who developed an OSCC (outcome); (2) patients with OE (population), with specific clinicopathological factors (exposure), in comparison with patients unexposed to those clinicopathological factors (comparison), and to determine their link to the MD of OE (outcome). Only longitudinal observational studies (type of studies) were used.

### 2.3 Search strategy

Electronic searches were carried out in MEDLINE via PubMed, EMBASE, Web of Science, Scopus, WHO regional bibliographic database LILACS, and the Conference Proceedings Citation Index databases. Searches combined thesaurus (e.g., MeSH and EMTREE) and free terms to maximize sensitivity. The algorithms used in each database contained the following keywords: “erythroplakia,” “erythroplasia,” “malignant transformation,” “malignant development,” “malignization,” “carcinogenesis,” “oral cancer,” “oral squamous cell carcinoma,” “mouth neoplasm,” and “prognosis.” These words were subjected to syntax adaptation for each database. All of the databases were searched from inception to February 2022 (upper limit).

We performed a second search, introducing each keyword in an unstructured fashion to check whether every article on the topic was retrieved. Potentially relevant articles that any of the authors were already familiar with and reference lists from the retrieved articles were also comprehensively checked. All retrieved references were managed using Mendeley v.1.19.9 (Elsevier, Amsterdam, The Netherlands).

### 2.4 Eligibility criteria

Criteria for eligibility of the studies included in the qualitative and quantitative analysis were as follows: (1) original research articles published in English language, (2) longitudinal observational studies, and (3) studies that analyses the MD of OE. The exclusion criteria were as follows: (1) letters to the editor, case reports, conference abstracts, clinical trials, etc.; (2) previous systematic reviews and meta‐analysis; (3) studies which reported on patients with diagnosis of erythroleukoplakia; (4) studies without a complete clinicopathological diagnosis of OE; and (5) studies that did not report specific data on OE.

### 2.5 Data selection and extraction

Articles were selected in two phases by two authors (APJ and MPS), first by screening titles and abstracts that apparently meet inclusion criteria, and then by reading the full texts to assess their final inclusion. During the calibration exercise, reviewers thoroughly discussed the criteria and applied them to a sample of 50% of the retrieved studies to determine the inter‐examiner agreement. After adequate agreement was achieved (*κ* score = 0.78), all of the studies were independently read by the reviewers. Any discrepancies between the researchers were resolved by a third or fourth researcher blinded to the study hypothesis (ILIM and JMA).

The information was retrieved by two investigators (APJ and MPS) using a custom‐made extraction sheet. The recorded data included: the first author and year of publication, country, study design, sample size, gender and age of patients, tobacco/alcohol/betel consumption, number and location of OE, histopathological data, follow‐up period, number of MD cases, and time until MD.

### 2.6 Statistical analysis

In the main meta‐analysis, we computed the prevalence MD events and that of total OE case by dividing the number of events by the sample size of the study. Then, we weighted the study‐specific log prevalence by the inverse of their variance to compute a pooled prevalence. For retrieved studies, the Clopper–Pearson interval was applied to estimate 95% confidence intervals (CIs). Combined pooled proportions were estimated with a random‐effect model (DerSimonian and Laird method). A further pre‐planned meta‐analysis to acknowledge the effect of clinicopathological factors on the MD of OE was considered, but this was discarded due to poor reporting of primary data. Therefore, we opted to perform a meta‐analysis of prevalence with the degrees of ED/presence of SCC in initial biopsy at diagnosis time‐point.

For statistical heterogeneity analysis, Cochran Q (*χ* ^2^) and Higgins *I* ^2^ test parameters were calculated. Cochran's Q test *p* < 0.1 was considered significant to assume apparent heterogeneity. The Higgins *I* ^2^ statistic cut‐off points of 25%, 50%, and 75% were considered to indicate low, moderate, and high heterogeneity, respectively.^13^

We assessed publication bias, first visually, using funnel plots, and then, more formally, using the test proposed by Egger et al.^14^ (performing a linear regression of the effect estimates on their standard errors, weighting by 1/[variance of the effect estimate], considering a *p*_Egger_ <0.1 as significant). The Metafor free R software package (v.3.6.2; https://www.r-project.org) was used for all statistical analysis as for figure plotting with user‐written commands. The significance level considered in all statistical analyses was 5% (*p* < 0.05).

### 2.7 Quality assessment and risk of bias

Risk of bias (RoB) was assessed using the Quality in Prognosis Studies–QUIPS tool, supported by Cochrane Prognosis Methods Group for prognosis studies. QUIPS considers the following domains: (1) study participation, (2) study attrition, (3) prognostic factor measurement, (4) outcome measurement, (5) study confounding, and (6) statistical analysis and reporting.^15^ RoB was qualified as low, moderate, or high for each domain. Each item scored as high adds 3 points, while when scored as moderate 2 and as low 1 to overall quality assessment for each study. Studies were categorized as high quality when the overall score was >13. RoB was assessed by two authors (MPS and APJ). Discrepancies between authors were resolved by all participating authors by consensus.

## 3 RESULTS

### 3.1 Bibliographical research

We identified 974 registers through the aforementioned search, whose abstracts were reviewed for contents relevant to the topic of this study, resulting in 633 exclusions. A total of 341 papers were then retrieved and, after careful consideration, 318 of them were excluded because their information was not useful for this study. The remaining 23 (2.37%) registers were checked according to the inclusion/exclusion criteria set for this investigation.

After the critical analysis of the studies, 10 (1.03%) studies from different geographical areas met the inclusion criteria: USA,^16^, ^17^ Denmark,^18^, ^19^ Brazil,^20^, ^21^ Taiwan,^22^, ^23^ China,^24^ and Thailand^25^ (Figure S1).

### 3.2 Malignant development

Only five studies^18^, ^19^, ^22^, ^23^, ^24^ stated the follow‐up period of patients. In these, mean MD rate was 12.7%, with a mean follow‐up period of 6.66 years (Table 1). In the three studies that reported cases of OE with MD,^19^, ^23^, ^24^ mean MD rate was 21.2%, with a mean follow‐up period of 8.25 years and a mean time until MD of 3.17 years. Conversely, in the two studies that did not report cases of MD, mean follow‐up period was 5.07 years^18^, ^22^ (Table 1).

**TABLE 1:** Main clinicodemographic data of patients with oral erythroplakia included in the study

Author and year	Country	Patients				Risk factors (*n*)			Follow‐up (years)	Malignant development (%)
		*n*	Gender		Age (years)	Tabaco	Betel	Alcohol		
			F	M						
Shafer and Waldron, 1975	USA	58	27	31	47 (>50) 11 (<50)	–	–	–	–	–
Nielsen et al. 1996	Denmark	10	9	1	–	–	–	–	6.3	0
Qin et al. 1999	USA	24	11	13	Mean: 61.9	15	–	–	–	–
Holmstrup et al. 2006	Denmark	15	–	–	–	5	–	–	7.5	6.67
Lapthanasupkul et al. 2007	Thailand	9	3	6	3 (<50) 6 (>50)	–	–	–	–	–
Feng et al. 2012	China	34	18	16	Mean: 58.7	10	–	12	16	50
Queiroz et al. 2014	Brazil	11	7	4	4 (<50) 7 (>50)	9	–	3	–	–
Yang et al. 2015	Taiwan	84	10	74	Mean: 54.2	65	57	42	3.83	0
Chuang et al. 2018	Taiwan	188	0	188	Mean: 46.0	170	160	120	9	6.9
de Azevedo et al. 2020	Brazil	8	5	3	61–80	1	–	1	–	–
*Total*		441	90	336	–	275	217	178	6.66 (mean)	12.7 (mean)

*Note*: F, female; M, male.

In the five studies without a follow‐up period of patients,^16^, ^17^, ^20^, ^21^, ^25^ mean MD of OE was 51.10%. Besides, 68.38% of cases of these studies were already SCC at the time of diagnosis.

### 3.3 Clinicopathological data

In this review, 90 women (20.4%) and 336 men (79.6%) with OE lesions were analyzed, mostly over 50 years^17^, ^21^, ^22^, ^24^, ^25^; (Table 1). From these, 75.55%, 56.31%, and 79.78% consumed tobacco, alcohol, and betel, respectively^17^, ^19^, ^20^, ^21^, ^22^, ^23^, ^24^ (Table 1).

Two studies^16^, ^22^ described patients with more than one OE. The most frequent location of OE was the buccal mucosa (38.5%), followed by the tongue (15.8%), the floor of the mouth (FOM) (12.3%), the gingiva (12.3%), the soft palate (11.2%), the retromolar area (6.9%), and the lip (2.7%) (Table 2).

**TABLE 2:** Location of oral erythroplakia lesions at the time of diagnosis and malignant development

Author and year	Oral erythroplakia (Time of diagnosis)							Oral Carcinoma (Malignant development)						
	FOM	Retromolar	Gingiva	Soft palate	Tongue	Buccal	Lip	FOM	Retromolar	Gingiva	Soft palate	Tongue	Buccal	Lip
Shaffer and Waldron, 1975	19	13	12	8	8	5	0	18	12	9	6	6	2	0
Nielsen et al. 1996	2	0	0	0	2	5	0	1	0	0	0	0	1	0
Qin et al. 1999	5	0	5	9	5	0	0	2	0	2	2	4	0	0
Holmstrup et al. 2006	0	0	3	0	1	11	0	–	–	–	–	–	–	–
Lapthanasupkul et al. 2007	1	0	5	0	0	3	0	–	–	–	–	–	–	–
Feng et al. 2012	0	0	4	5	16	9	0	0	–	–	3	10	4	–
Queiroz et al. 2014	2	1	1	2	3	0	2	–	–	–	–	–	–	–
Yang et al. 2015	2	4	1	2	5	65	5	–	–	–	–	–	–	–
de Azevedo et al. 2020	1	0	1	3	1	2	0	–	–	–	–	–	–	–
Total	32	18	32	29	41	100	7	21	12	11	11	20	7	0

Abbreviation: FOM, floor of the mouth.

Regarding the presence of ED, different evaluation systems were used; thus, we divided ED into low‐grade ED (previous mild to moderate) and high‐grade ED (previous moderate to severe), following the WHO proposal.^26^ In the initial biopsy, 47.9% of cases showed ED: 76.6% low‐grade ED and 23.4% high‐grade ED.^16^, ^17^, ^18^, ^19^, ^20^, ^21^, ^22^, ^24^ A total of 42.8% of the OE corresponded to SCC at the initial biopsy (Table 3).

**TABLE 3:** Histopathological data of the oral erythroplakia at the time of diagnosis

Author and year	*n*	SCC	High‐risk ED	Low‐risk ED	No ED
Shaffer and Waldron, 1975	65	59	0	6	0
Nielsen et al. 1996	9	2	0	4	3
Qin et al. 1999	24	10	8	6	0
Holmstrup et al. 2009	15	2	2	10	1
Lapthanasupkul et al. 2007	9	3	0	3	3
Feng et al. 2012	34	0	14	20	0
Queiroz et al. 2014	11	3	5	3	0
Yang et al. 2015	84	23	0	41	20
de Azevedo et al. 2020	8	5	0	2	1
Total	250	107	29	95	28

Abbreviations: ED, epithelial dysplasia; OE, oral erythroplakia; SCC, squamous cell carcinoma.

Only Feng et al.^24^ reported the clinical data of patients who underwent MD: 8 females and 9 males, all of them older than 50 years. Also, 42% and 50% of patients were tobacco and alcohol consumers, respectively.^17^, ^24^ The most common area of MD was the FOM (25.6%), followed by the tongue (24.3%), the retromolar area (14.6%), the gingiva (13.4%), the soft palate (13.4%), and the buccal mucosa (8.5%). Only Feng et al.^24^ stated that 47.1% of cases with MD showed high‐grade ED and 52.9% low‐grade ED on the initial diagnostic biopsy.

### 3.4 Meta‐analysis

**FIGURE 1:**
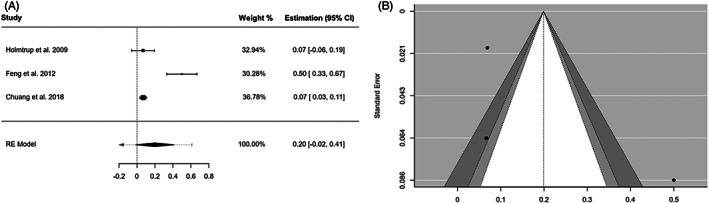
Malignant development. (A) Forest plot representing the meta‐analysis of the malignant development rate of oral erythroplakia. RE (random‐effects) and weight of each study. (B) Funnel plot assessing the publication bias

In the main meta‐analysis, three studies^19^, ^23^, ^24^ testing the same hypothesis (prevalence of MD in OE) were included in the quantitative synthesis. The combined MD rate was 19.9% (95% CI = −1.6 to 41.4; *I* ^2^ = 91.74%, *p*‐value by Q test = 0.00001) (Figure 1A). A remarkable asymmetry with a lack of studies on the middle of the plot was seen (Figure 1B); nonetheless, Egger's regression test neglected the existence of publication bias (*p*‐value of the intercept = 0.276).

**FIGURE 2:**
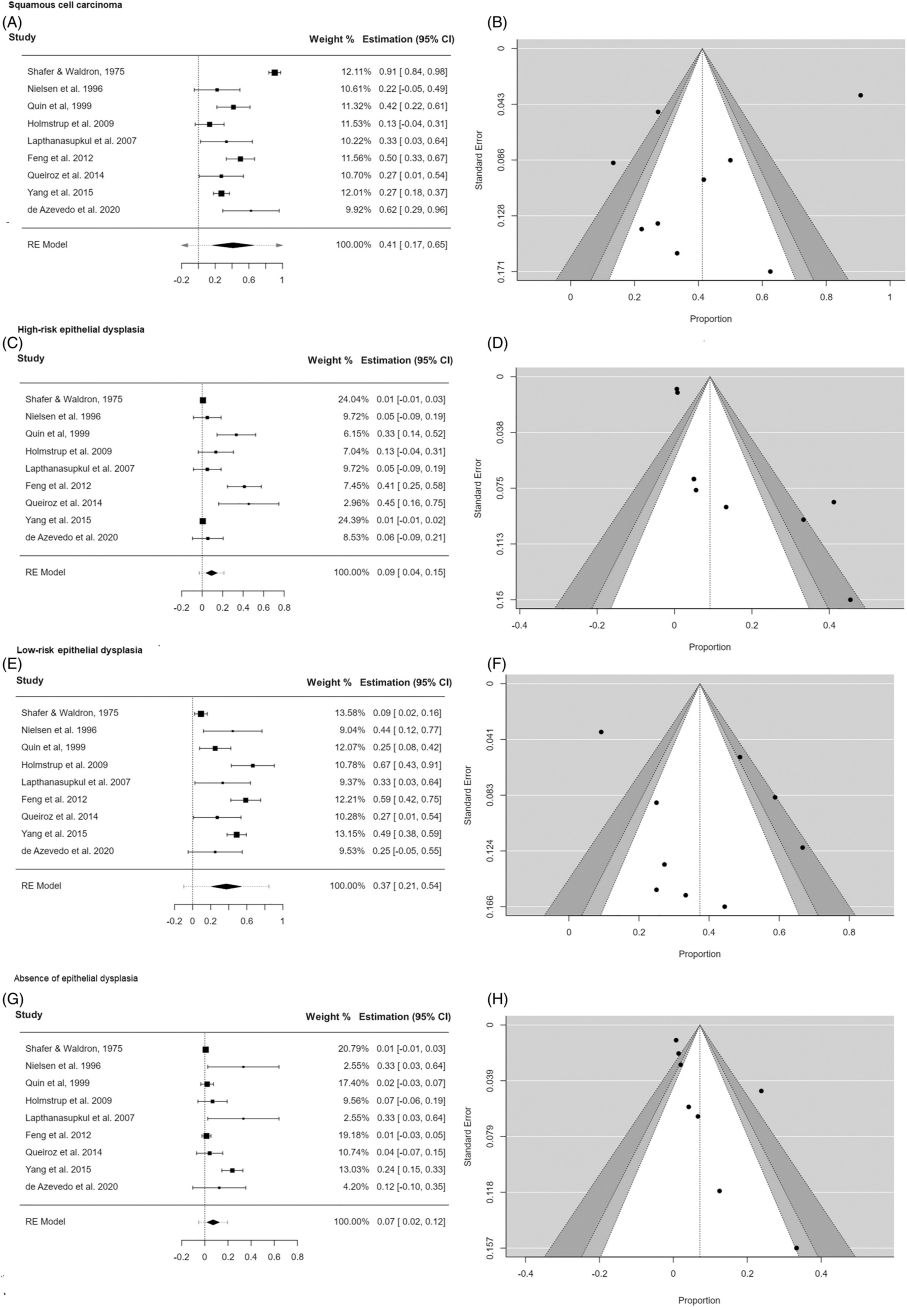
Initial histopathological diagnosis. Forest plot of the initial histopathological diagnosis of the oral erythroplakias that suffer malignant development. Funnel plot to assess the publication bias. (A & B): Squamous cell carcinoma; (C & D): High‐risk epithelial dysplasia; (E & F): Low‐risk epithelial dysplasia; (G & H): Absence of epithelial dysplasia

In the secondary analysis, we performed a pooled analysis on the prevalence of initial histopathological diagnosis: SCC, high‐grade ED, low‐grade ED, and absence of ED (Table 4). Globally, studies harbored a substantial degree of heterogeneity and no individual study seemed to represent an influential point that increased heterogeneity dramatically. Presence of ED was almost six times higher than absence of ED (41.2% vs. 7.19%). Observation of the asymmetry of the funnel plots (Figure 2) and the statistical tests conducted for the same purpose confirmed the absence of “small‐study” effects on the pooled prevalence of the initial diagnosis of SCC (*p*_Egger_ = 0.549) and low‐grade ED (*p*_Egger_ = 0.685). The exceptions were high‐grade ED (*p*_Egger_ = 0.001) and absence of ED (*p*_Egger_ = 0.002), for which bias could not be ruled out.

**TABLE 4:** Pooled prevalence, MD rate, and subgroup analysis of the initial histopathological diagnosis of OE

	Sample size (*n*)		Pooled data		Heterogeneity	
	Studies	Patients	ES (95% CI)	*p*‐value	*p*_het_	*I* ^*2*^ (%)
Malignant development						
	3	237	PP = 19.9% (−1.6–41.4)	0.001	0.0001	91.7
Initial histopathological diagnosis						
SCC	9	259	PP = 41.2% (16.8–65.5)	0.001	0.0001	95.2
High‐risk ED	9	259	PP = 9.17% (29.8–51.4)	0.001	0.0001	82.5
Low‐risk ED	9	259	PP = 37.4% (20.9–53.9)	0.001	0.0001	87.9
No ED	9	259	PP = 7.19% (2.0.12.4)	0.007	0.0001	75.63

Abbreviations: CI, confidence intervals; CIS, carcinoma in situ; ED, epithelial dysplasia: OE, oral erythroplakia; PP, pooled proportion; SCC, squamous cell carcinoma.

### 3.5 Risk of bias

The QUIPS tool showed that RoB was high in three studies^16^, ^17^, ^18^ and low in seven (18–24] (Figure S2). The most potential biases were inappropriate statistical analyses and poor assessment of study cofounding. Nevertheless, the quality of some studies was also sub‐optimal in the remaining four domains.

## 4 DISCUSSION

Erythroplakia is a premalignant disorder first described as a raised, velvety red plaque in the male and female genital mucosa.^1^, ^27^ The first oral cases were published in the late twentieth century,^28^, ^29^ rapidly becoming a matter of controversy.

In 1955, Blau & Hyman^30^ made the first review on OE, with lesions on the tongue, the buccal mucosa, and the lip. Later, Shear^31^ proposed three clinical forms of OE (homogeneous, erythroleukoplakia, and speckled), with different prognostic aspects; which were later approved by several authors.^10^, ^16^ In 2007, “erythroleukoplakia” was considered an OPMD independent of OE,^32^ appearing as such in the latest WHO Classification of Head and Neck Tumours.^26^ Recently, erythroleukoplakia has been once again reconsidered as a clinical form of nonhomogenous of OL,^9^ removing it as an individual OPMD.

Taking into account the current definition of OPMD: “*any oral mucosal abnormality that is associated with a statistically increased risk of developing oral cancer*”,^9^ we believe that some aspects of previous reviews about the MD of OE are somewhat questionable.^6^, ^33^ Most studies include, at the same time, cases of OE, erythroleukoplakia, and SCC.^34^, ^35^, ^36^, ^37^, ^38^, ^39^, ^40^ Furthermore, MD assessment of OE has been preferably based on Shafer & Waldron's^16^ classic study on nonhomogeneous OE, where 91% of cases were already SCC at the time of diagnosis. In order to avoid further confusions, we only selected cases clinicopathologically diagnosed as homogeneous OE for our review.

We think that clinical follow‐up of patients is necessary to assess the true MD of OE as an OPMD. Therefore, when we considered the studies without a follow‐up period, we achieved a “wrong” MD rate of 50.51%, similar to that of previous reviews (44.9%–50%).^6^, ^33^ On the contrary, when we considered the studies with a follow‐up period, we obtained a “right” MD rate of 19.9%. Moreover, the number of SCC in the studies without a clinical follow‐up (68.38%) is two times bigger than in all the studies (30.99%), and five times bigger than in the studies with a clinical follow‐up (12.7%) (Table 1).

All these findings question whether OE is the OPMD with the highest risk of MD, as previously stated.^9^, ^32^ We believe that OE should only be considered as an OPMD when (1) there is a clinical follow‐up period of patients, and (2) the initial biopsy rules out the presence of an SCC. Since the follow‐up period has been longer in the studies with cases of MD (8.25 vs. 5 years) (Table 1), we think patients with OE should be actively followed for life.

Except for the study by Chuang et al.,^23^ gender and age distribution were similar in patients with OE and those who suffer MD (Table 1).^41^, ^42^ The buccal mucosa has been the most frequent location of OE; which could be related to some particular carcinogenic habits, such as consumption of betel.^22^, ^25^ However, the absence of cases with MD in some studies^22^, ^25^ suggest that some of these cases may not be true OE.

We also observed interesting differences between the location OE and where MD occurred. Some infrequent areas of OE like the FOM, the tongue, and the retromolar area are common sites of MD (FOM: 25.6%, tongue: 24.3%, and retromolar area: 14.6%). In contrast, more frequent areas of OE like the buccal mucosa and the lip have a low rate of MD (buccal mucosa: 8.5%, lip: 0%). These results suggest the existence of specific locations of the oral mucosa with a bigger risk for MD of OE. Also, they point to the need for a thorough clinical and histopathological differential diagnosis with all mucous pathologies clinically similar to OE, preferably SCC, but also oral lichenoid disease (OLD), erythematous candidiasis, lupus erythematosus, pemphigoid, and so forth.^9^ Performing a good clinicopathological correlation is key to reach the final diagnosis of OE; thus, it is mandatory to get a good biopsy of all suspicious oral “red plaques” in order to (1) rule out other diagnoses and (2) assess the existence and/or degree of ED.

In 1972, Shear^31^ proposed two histopathological types of OE: a “neoplastic” one, associated with SCC, carcinoma in situ or ED; and an “inflammatory” one, related to *Candida* infection, prosthetic stomatitis, immunological processes, and so forth. In our opinion, this classification is misleading and should not be used. Currently, we cannot consider as an OPMD an oral lesion that is already a SCC in the histopathological analysis; or can all “red plaque‐like” lesions that may appear on the oral mucosa be considered as an OE.^9^ Indeed, some multifocal cases of OE reviewed on this study,^22^ or infected with *Candida spp*., and without ED or low‐grade ED, could actually represent other disorders.

The histopathological diagnosis is the gold standard technique for the prognostic assessment of all OPMD, including OE. Different oral mucous pathologies of an inflammatory nature may cause epithelial changes similar to those seen in ED, making the final clinicopathological diagnosis very difficult.^43^ Only the study by Feng et al.^24^ assesses the presence of ED in the initial diagnostic biopsy of cases with MD, all of which showed ED (47.1% high grade and 52.9% low grade). In our review, almost half of the OE lesions had ED in the first biopsy, and presence of ED was six times higher than absence of ED (41.2% vs. 7.19%) (Table 4). Moreover, ED remains the most important prognostic factor in relation to the MD of multiple OPMD, such as OL, OLD, and proliferative multifocal/verrucous leukoplakia.^44^, ^45^, ^46^

Unfortunately, we had several limitations during the performance of this systematic review and meta‐analysis. First, is the low number of observational studies on the MD of OE, and with good clinical follow‐up period of patients; and second, is the lack of standardization and changes in the diagnostic criteria for OE over the years. This jeopardized the association between the MD of OE and different clinicopathological features.

## 5 CONCLUSIONS

In summary, overall MD rate of OE in this study was 19.9%, significantly lower than previously reported. We were unable to associate this MD with any specific clinicopathological feature, including ED. Nevertheless, we believe the presence of ED in the initial diagnostic biopsy remains the main prognostic factor in this OPMD. Further observational studies are needed, with well‐established diagnostic criteria and good clinical follow‐up, in order to identify the true risk of MD of OE and its related factors. This way we could design effective preventive and therapeutic programs.

## AUTHOR CONTRIBUTIONS

*Conceptualization*, *data curation*, *formal analysis*, *methodology*, *resources*, *software*, *validation*, *visualization*, *and writing‐original draft*: Alejandro Ismael Lorenzo‐Pouso. *Conceptualization*, *investigation*, *methodology*, *writing‐original draft*, *writing‐review & editing*: Irene Lafuente‐Ibáñez de Mendoza. *Conceptualization*, *data curation*, *formal analysis*, *methodology*, *project administration*, *resources*, *supervision*, *validation*, *writing‐review & editing*: Mario Pérez Sayáns *Data curation*, *investigation*, *methodology*, *writing‐original draft*: Alba Pérez‐Jardón. *Data curation*, *investigation*, *methodology*, *writing‐original draft*: Cintia M. Chamorro‐Petronacci. *Data curation*, *investigation*, *methodology*, *supervision*, *writing‐original draft*: Andrés Blanco‐Carrión. *Conceptualization*, *data curation*, *formal analysis*, *investigation*, *project administration*, *resources*, *supervision*, *validation*, *writing‐review & editing*: José Manuel Aguirre‐Urizar.

## FUNDING INFORMATION

This research did not receive any specific grant from funding agencies in the public, commercial, or not‐for‐profit sectors.

## CONFLICT OF INTEREST

Authors confirm there is no conflict of interest regarding the submission of this manuscript.

## Supporting information


**Figure S1** PRISMA flow diagram. Synthesis of the bibliographic analysis.

**Figure S2** RoB. Risk of bias of the included studies according to Quality in Prognosis Studies (QUIPS).

## Data Availability

The data that support the findings of this study are available from the corresponding author upon reasonable request.
