# Supplementary figures and images for: Critical update, systematic review, and meta‐analysis of oral erythroplakia as an oral potentially malignant disorder
Source: J Oral Pathol Med. 2022 May 12;51(7):585–93. doi: 10.1111/jop.13304 (PMC9545979; doi:10.1111/jop.13304)

**Supplementary figure 1.** **PRISMA flow diagram.** Synthesis of the bibliographic analysis.


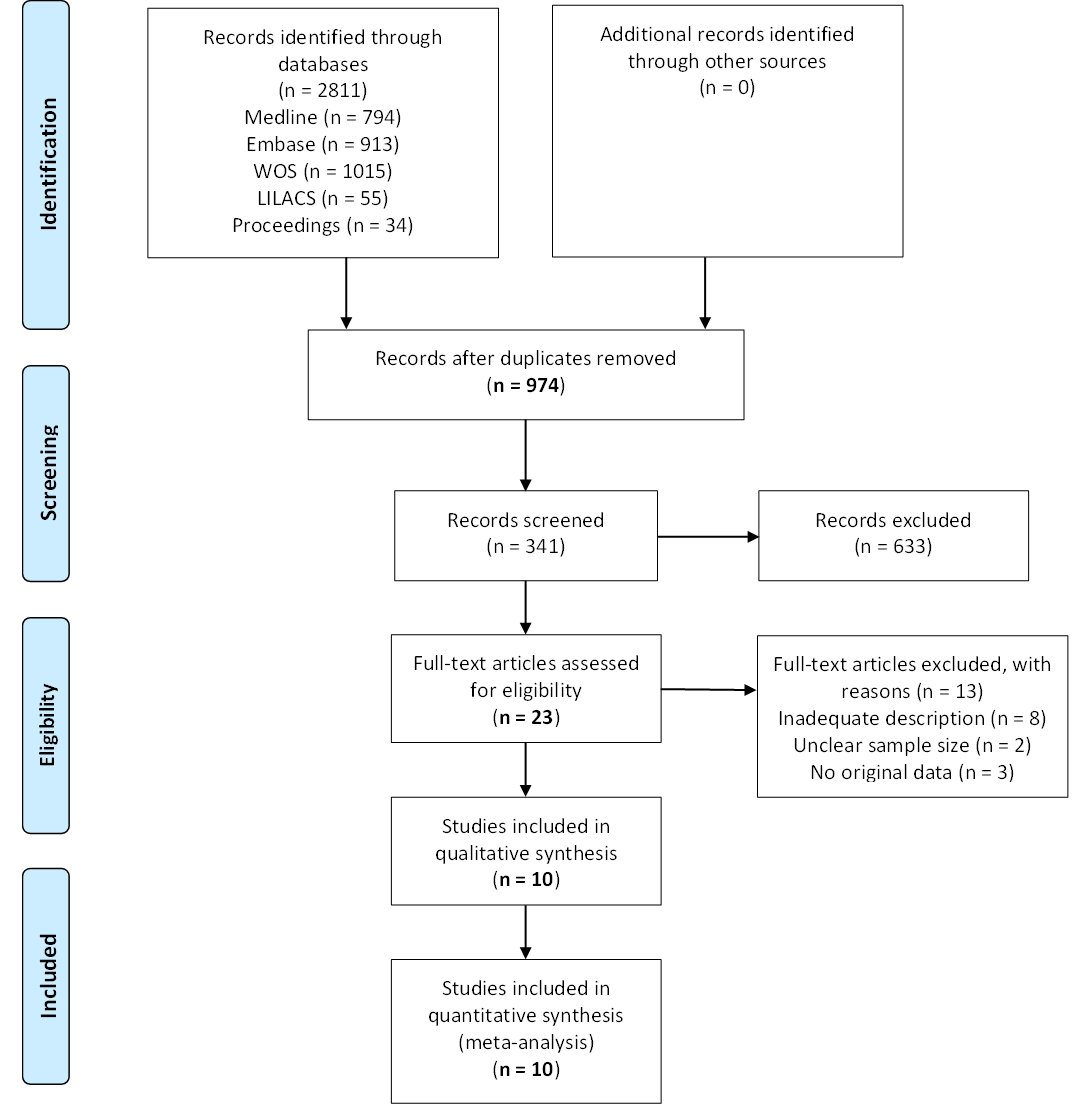

Supplement: Supplementary file 1 — Figure S1 PRISMA flow diagram. Synthesis of the bibliographic analysis. [file JOP-51-585-s002.docx]
