# Supplementary material for: Critical update, systematic review, and meta‐analysis of oral erythroplakia as an oral potentially malignant disorder
Source: J Oral Pathol Med. 2022 May 12;51(7):585–93. doi: 10.1111/jop.13304 (PMC9545979; doi:10.1111/jop.13304)

**Supplementary figure 2. RoB.** Risk of bias of the included studies according to Quality in Prognosis Studies (QUIPS).


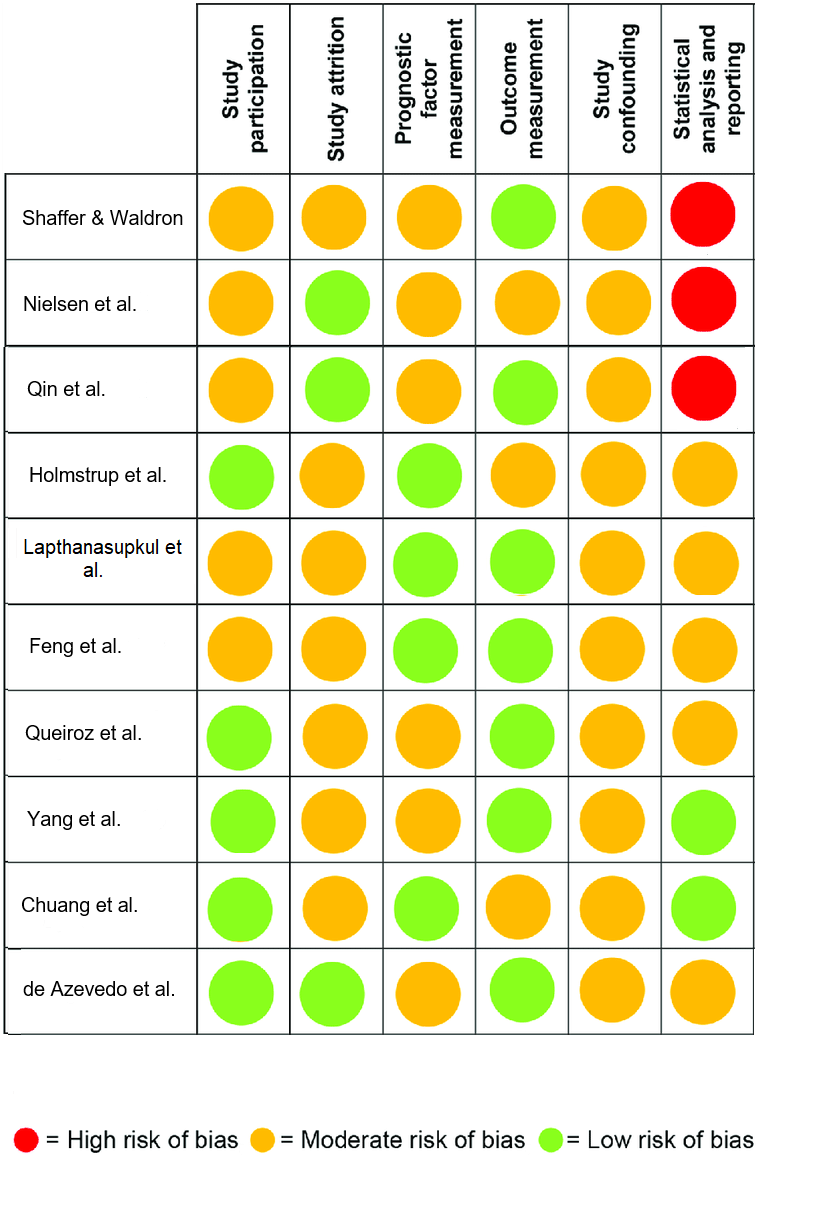

Supplement: Supplementary file 2 — Figure S2 RoB. Risk of bias of the included studies according to Quality in Prognosis Studies (QUIPS). [file JOP-51-585-s001.docx]
